# Simulated data and code for analysis of herpetofauna response to forest management in the Missouri Ozarks

**DOI:** 10.1016/j.dib.2016.08.043

**Published:** 2016-08-30

**Authors:** Christopher T. Rota, Alexander J. Wolf, Rochelle B. Renken, Robert A. Gitzen, Debby K. Fantz, Robert A. Montgomery, Matthew G. Olson, Larry D. Vangilder, Joshua J. Millspaugh

**Affiliations:** a West Virginia University, School of Natural Resources, United States; b University of Missouri, Fisheries and Wildlife Sciences, United States; c Missouri Department of Conservation, United States; d Auburn University, School of Forestry and Wildlife Sciences, United States; e Michigan State University, Department of Fisheries and Wildlife, United States

## Abstract

We present predictor variables and R and Stan code for simulating and analyzing counts of Missouri Ozark herpetofauna in response to three forest management strategies. Our code performs four primary purposes: import predictor variables from spreadsheets; simulate synthetic response variables based on imported predictor variables and user-supplied values for data-generating parameters; format synthetic data for export to Stan; and analyze synthetic data.

**Specifications Table**

**Table:** 

Subject area	Biology
More specific subject area	Wildlife Biology, Herpetology, Forest Ecology
Type of data	Table, supplementary files
How data was acquired	Data in PRISM.csv was obtained from, or calculated as a function of, data obtained from the PRISM Climate Group [6]; data in Drought.csv was obtained from the National Centers for Environmental Information [5]; data in Treatment.csv and Time Since Disturbance.csv are the treatment levels and time since treatment, respectively, at the Missouri Ozark Forest Ecosystem Project (Rota et al., in press); data in array_covs.csv are array-level covariates and include landform position and soil type, which were classified according to Meinert et al. [4], and northeastness, which was measured as aspect with a 10 m digital elevation model and transformed following Beers et al. [2].
Data format	Filtered, simulated, formatted for analysis
Experimental factors	Sites were treated with even-aged management techniques (clearcut, intermediate thin), uneven-aged management techniques (group opening, single-tree selection) and no-harvest management techniques (leave)
Experimental features	We used a completely randomized block design. We had 3 blocks, each which were subdivided into 3 forest compartments. Within each block, each compartment was randomly assigned even-aged, uneven-aged, or no-harvest management treatments.
Data source location	Carter, Shannon, and Reynolds County, Missouri, USA
Data accessibility	Data is with this article

**Value of the data**

• Simulating data with known data-generating parameters, and subsequently fitting a model to those data, allows researcher to evaluate how well our statistical model recovers known parameters.
• Presenting our code allows other researcher to replicate the analyses presented in Rota et al. (In Press), or to adapt our code for similar projects.
• Long-term experimental studies allow researchers to draw strong inference regarding the effects of forest management on reptiles and amphibians.

## 1 Data

The data we report includes a mix of covariates measured in the field and synthetic counts of reptiles and amphibians simulated from known covariates and user-supplied values of data-generating parameters. Data import, simulation, and analysis can all be completed by executing commands in the file “model.R”. Make sure all files are saved together in the working directory and the “gdata” and “rstan” packages are properly installed in program R (R Core Development Team [7]). “model.R” includes code for inputting “design matrices.R”, which imports and manipulates covariate data stored in spreadsheets (array_covs.csv, Drought.csv, PRISM.csv, Time Since Disturbance.csv, and Treatments.csv). “model.R” also includes code for inputting “simulating data.R”, which simulates count data based on observed covariates and user-supplied data-generating values, and formats data for export to Stan. “model.stan” specifies the statistical model that is run by program Stan (Carpenter et al. [3]).

## 2 Experimental design, materials and methods

Details of the experimental design are presented in Rota et al. [1]. We were unable to publish data on observed counts of reptiles and amphibians at the time of publication. We therefore simulated counts of reptiles and amphibians based on observed values of covariates and user-specified data-generating parameters. The code for simulating synthetic count data, as well as the values of the data-generating parameters, are specified in “simulating data.R”.
